# Measuring Health-Related Quality of Life of HIV-Positive Adolescents in Resource-Constrained Settings

**DOI:** 10.1371/journal.pone.0040628

**Published:** 2012-07-16

**Authors:** Caroline Masquillier, Edwin Wouters, Jasna Loos, Christiana Nöstlinger

**Affiliations:** 1 Department of Sociology, Research Centre for Longitudinal and Life Course Studies (CELLO), University of Antwerp, Antwerp, Belgium; 2 Department of Public Health, ITM HIV/AIDS Center (IHAC), Institute of Tropical Medicine (ITM), Antwerp, Belgium; London School of Hygiene and Tropical Medicine, United Kingdom

## Abstract

**Background and Objectives:**

Access to antiretroviral treatment among adolescents living with HIV (ALH) is increasing. Health-related quality of life (HRQOL) is relevant for monitoring the impact of the disease on both well-being and treatment outcomes. However, adequate screening tools to assess HRQOL in low-resource settings are scarce. This study aims to fill this research gap, by 1) assessing the psychometric properties and reliability of an Eastern African English version of a European HRQOL scale for adolescents (KIDSCREEN) and 2) determining which version of the KIDSCREEN (52-, 27- and 10-item version) is most suitable for low-resource settings.

**Methods:**

The KIDSCREEN was translated into Eastern African English, Luganda (Uganda) and Dholuo (Kenya) according to standard procedures. The reconciled version was administered in 2011 to ALH aged 13–17 in Kenya (n = 283) and Uganda (n = 299). All three KIDSCREEN versions were fitted to the data with confirmatory factor analysis (CFA). After comparison, the most suitable version was adapted based on the CFA outcomes utilizing the results of previous formative research. In order to develop a general HRQOL factor, a second-order measurement model was fitted to the data.

**Results:**

The CFA results showed that without adjustments, the KIDSCREEN cannot be used for measuring the HRQOL of HIV-positive adolescents. After comparison, the most suitable version for low-resource settings - the 27-item version - was adapted further. The introduction of a negative wording factor was required for the Dholuo model. The Dholuo (CFI: 0.93; RMSEA: 0.039) and the Luganda model (CFI: 0.90; RMSEA: 0.052) showed a good fit. All cronbach’s alphas of the factors were 0.70 or above. The alpha value of the Dholuo and Lugandan HRQOL second-order factor was respectively 0.84 and 0.87.

**Conclusions:**

The study showed that the adapted KIDSCREEN-27 is an adequate tool for measuring HRQOL in low-resource settings with high HIV prevalence.

## Introduction

Adolescents and young adults are at the epicenter of the global HIV epidemic [1]. Globally, young people (aged 15–24) accounted for 41% of new infections among persons aged 15 and older in 2009, with 79% of these new infections occurring in sub-Saharan Africa. In Uganda and Kenya – the geographical areas of this study –46000 and 42000 new HIV infections were reported among adolescents in 2009 (aged 15–24) [2]. As do their uninfected counterparts, adolescents living with HIV/AIDS (ALH) struggle with the biologic, cognitive and social developmental challenges related to adolescent transition [3], [4], [5], [6], [7], but growing evidence suggests that ALH are also confronted with the challenges of living with a chronic disease which is potentially fatal and socially stigmatizing, e.g. coping with HIV-stigma, and adopting preventive behaviors [8].

One main element of policies to mitigate the large HIV-burden in those areas has been public-sector delivery of antiretroviral treatment (ART). There is widespread empirical evidence of the effectiveness of pediatric ART programs in resource-limited settings. In the absence of a cure, HIV-infected adolescents will need to adhere to ART on a lifelong basis, which is a prerequisite for their survival [9], [10]. For adults, it has been shown that health-related quality of life (HRQOL) and adherence influence each other [11], [12]. Consensus is growing regarding the positive impact of HRQOL when adults in the West [13] and in Africa [14] adhere to ART. Therefore, HRQOL information is relevant for monitoring both the impact of the disease on individual well-being and to measure treatment outcomes [15].

Many studies have addressed the challenges of improving HRQOL among HIV positive adults. However, little research has described HRQOL of HIV-positive adolescents in Sub-Saharan Africa after the introduction of ART in the public sector [16]. In order to assess the HRQOL of adolescents in countries with a high HIV-burden, a good quality measure is indispensable. A review of the literature revealed no suitable instrument. A number of HRQOL measures have been developed and used for adolescents in Western countries, such as AUQUEI, HI, QUALIN, CHIP-AE, CHQ and KIDSCREEN [17], [18]. To be meaningful, it is important that a HRQOL measure is culturally appropriate, age-specific and designed for adolescents living with a chronic disease [15]. However, none of these instruments has been tested in resource-constrained settings with a high prevalence of HIV.

The current study aims to address this research gap by testing the reliability of an Eastern African adaptation of the KIDSCREEN questionnaire. We selected this European HRQOL scale for adolescents for several reasons. First, this questionnaire is a truly cross-national HRQOL measure, since it was simultaneously developed in 13 European countries [19]. Its cross-cultural foundation makes it an interesting questionnaire when testing its appropriateness in two Sub-Saharan resource-constrained settings. Previous studies have successfully adapted the extensive version of this measurement instrument to Korean [20] and Brazilian [21] populations. Second, this instrument was specifically developed for children and adolescents with a chronic disease, which is preferable over general HRQOL measures [22]. Although not designed specifically for HIV-positive adolescents, this generic instrument integrates consequences of co-morbidities and potential side effects from treatment into one single assessment. An HIV-specific instrument would need to address the HRQOL impact of all opportunistic infections, which would lead to scales with a lot of items [15]. Third, our literature review revealed that the KIDSCREEN is based on a more comprehensive definition of adolescents’ HRQOL in comparison with other measures. Some of the existing measurement instruments focus solely on physical and psychological wellbeing [3], [23], whereas social factors are also very important for adolescents living with HIV/AIDS [18]. The KIDSCREEN questionnaire includes various latent factors concerning social life, physical and psychological wellbeing. Furthermore, it also encompasses factors regarding self-perception and autonomy. Fourth, the KIDSCREEN offers three different versions: an extensive version with 52 items (KIDSCREEN-52), a condensed version with 27 items (KIDSCREEN-27) and a short version with ten items (KIDSCREEN-10). Because health care personnel works with limited resources - particularly in settings with HIV prevalence - a brief and reliable measurement instrument is indispensable [24], [25]. Therefore, these different versions provide the opportunity to test and adapt the most condensed questionnaire possible.

The objectives of this study are two-fold: 1) to assess the reliability of the Eastern African adaptation of this HRQOL scale for adolescents (KIDSCREEN) and 2) to determine which version of the KIDSCREEN (the 52, 27 or 10 items) is most suitable for low-resource settings.

## Methods

### Ethics Statement

The study’s purpose and all the procedures involved were explained in a youth-friendly and understandable way to all potential participants and their caregivers. After assessing whether they had understood the benefits and risks involved in participating in the study, parental (or guardian) informed written consent and assent from adolescents was obtained before participation [26] in each step of the study.

The study obtained ethical approval from the institutional review boards (IRB) of all institutions involved; in Kenya the IRB of Kisumu Kenya Medical Research Institute (KEMRI), and in Nairobi KEMRI and the National Ethics Review Committee (Nairobi). For Uganda, we obtained approval from Makerere University College of Health Sciences, the Uganda National Council of Science and Technology and from Baylor College of Medicine (Texas, US). In Belgium, approval was obtained at the Institute of Tropical Medicine’s IRB, and the Committee for medical ethics at the University Hospital Antwerp.

### Setting

In Kenya, participants were recruited from three areas in Nyanza province, namely Asembo and Gem, two rural communities along the shores of lake Victoria and Kisumu, the capital of Nyanza. With 5.8% of adolescents aged 15–19 years living with HIV, this region is the most affected of all Kenyan provinces [26], [27]. The Ugandan study setting, Kampala municipality, is also located in a region with the highest HIV prevalence (8.5% by 15–49 years old) in Uganda [28].

### Sample and Study Population

This study was undertaken within the framework of the development and evaluation of a sexual and reproductive health (SHR) intervention for adolescents living with HIV, entitled ‘Positive Living for a Brighter Future’ (BF). The cross-cultural adaptation and translation of the HRQOL instrument was part of the preparation of the baseline assessment for this study. The sample size calculation was based on the proportion of HIV-positive adolescents (aged 13–17 years) who reported having had sex in the previous 3 months, since this behavioral characteristic was most relevant in the planned outcome evaluation of the BF intervention [26].

Respondents were recruited from participating sites that provide regular HIV care and follow-up in Kenya and Uganda. In Kenya, support centers and support groups of the communities described above participated in the study. In Uganda, participants were recruited from the Pediatric Infectious Disease Clinic (PIDC) in Kampala. Inclusion criteria were: voluntary study participation; aged between 13–17 years; never married or co-habitating; resident in the study area; parental (or guardian’s) informed written consent to participate; able to assent; aware of HIV sero-status for at least 3 months.

### Development of the Eastern African Version of the KIDSCREEN and Translation into Dholuo and Luganda

The original European KIDSCREEN-52 consists of both positively and negatively worded items, each rated on a five-point Likert scale (e.g. ranging from never to always). The 27- and 10-item questionnaire are condensed versions using the same items. Table 1 shows the questions from the translated KIDSCREEN versions with respectively 52, 27 and 10 items. The KIDSCREEN-52 measures HRQOL in ten dimensions covering *physical activities and health* (1), *feelings* (2), *general mood* (3), *self-perception* (4), *free time* (5), *family and home life* (6), *financial resources* (7), *friends* (8), *school and learning* (9), *social acceptance* (10). The KIDSCREEN-27 has five latent concepts: *physical activities and health* (1), *general mood and feelings* (2–3), *family and free time* (5–6), *friends* (8), *school and learning* (9). The most condensed version, the KIDSCREEN-10, consists of one latent factor, i.e. *HRQOL* (11).

**Table 1:** The approved Eastern African translation of the KIDSCREEN.

Factor	No.	Question	Number of items		
		Thinking about the last week…	52	27	10
Physical activities and health	1	In general, how would you say your health is?	1	1	
	2	Did you feel physically fit?	1	1	11
	3	Were you physically active (e.g. running around, physical exercises, sports)?	1	1	
	4	Were you able to do these physical exercises well?	1	1	
	5	Did you feel full of energy?	1	1	11
Feelings	6	…, did you enjoy your life?	2	2–3	
	7	Did you feel happy that you are alive?	2		
	8	Did you feel satisfied with your life (happy and content)?	2		
	9	Were you in a good mood?	2	2–3	
	10	Did you feel joyful?	2		
	11	Did you have fun?	2	2–3	
General mood	12	…, did you feel that you do everything badly?	3		
	13	Did you feel sad?	3	2–3	11
	14	Did you feel so bad that you didn’t want to do anything?	3	2–3	
	15	Did you feel that everything in your life goes wrong?	3		
	16	Did you feel fed up?	3		
	17	Did you feel lonely?	3	2–3	11
	18	Did you feel under pressure?	3		
Self- Perception	19	…, did you feel happy with the way you are?	4	2–3	
	20	Did you feel happy with your clothes?	4		
	21	Were you worried about the way you look?	4		
	22	Did you feel jealous of the way other girls/boys look?	4		
	23	Would you have liked to change something about your body?	4		
Free time	24	…, did you have enough free time for yourself?	5	5–6	11
	25	Were you able to do the things that you wanted to do in your free time?	5	5–6	11
	26	Did you have enough chances to go outdoors?	5		
	27	Did you have enough time to meet friends?	5		
	28	Were you able to choose what to do in your free time?	5		
Family and home life	29	…, did parent(s)/guardian(s) understand you?	6		
	30	Did you feel loved by your parent(s)/guardian(s)?	6		
	31	Were you happy at home?	6		
	32	Did your parent(s)/guardian(s) have enough time for you?	6	5–6	
	33	Did your parent(s)/guardian(s) treat you fairly?	6	5–6	11
	34	Were you able to talk to your parent(s)/guardian(s)? when you wanted to?	6	5–6	
Financial resources	35	…, did you have enough money to do the same things as your friends?	7	5–6	
	36	Did you have enough money for things you needed last week?	7	5–6	
	37	Did you have money to do things with your friends?	7		
Friends	38	…, did you spend time with your friends?	8	8	
	39	Did you do activities with other girls and boys?	8		
	40	Did you have fun with your friends?	8	8	11
	41	Did you and your friends help each other?	8	8	
	42	Were you able to talk about everything with your friends?	8		
	43	Were you able to depend on your friends?	8	8	
School and learning	44	…, were you happy at school?	9	9	
	45	Were you getting along well at school?	9	9	11
	46	Were you happy with your teachers?	9		
	47	Were you able to pay attention in class?	9	9	11
	48	Did you enjoy going to school?	9		
	49	Did you get along well with your teachers?	9	9	
Social acceptance	50	…, were you afraid of other girls and boys?	10		
	51	Did other girls and boys tease you?	10		
	52	Did other girls and boys harass you?	10		

We translated the original English KIDSCREEN-52 conform to the KIDSCREEN translation and validation procedure issued by the KIDSCREEN group into the local languages of the study populations: Dholuo for the Kenyan subsample and Luganda for the Ugandan subsample. This included the following steps: forward translation by two independent translators. After having reached consensus on problematic items, one reconciled forward translation in the target languages was issued, and translated backward into English by a third independent translator. Forward and backward translations were then reviewed resulting in a final forward translation. Next, the conceptual equivalence was assessed through a telephone conference with the research teams involved, resulting in harmonized versions, which were subjected to cognitive pre-tests carried out in pilot face-to-face interviews with six adolescents per language version to assess the comprehensibility and acceptability of the translated items. The linguistic and conceptual problems revealed at this stage were discussed between all researchers involved. Problems in understanding specific items referred to contextual differences (e.g. biking or climbing are not a common leisure time activities; children usually do not receive pocket money and so have little money at their own disposition; having free time was an unfamiliar concept) or to the secondary meaning of specific terms (e.g. ‘satisfied’ was understood as ‘being full’ mainly in relation to food intake). To maintain the coherence of the KIDSCREEN, the questions on *financial resources* and *free time* were kept in the adapted version, but were marked as a point for further exploration in the baseline study. Based on this consensus, final harmonized versions in Dholuo and Luganda and their respective adaptation of the Eastern African English version, containing some linguistic differences from the UK English version, were compiled.

### Data Collection Procedures

Data were collected through trained interviewers who conducted face-to-face interviews using a standardized questionnaire that included the KIDSCREEN and other questions relevant to living with HIV and sexual and reproductive health. Although English is an official second language in both countries and commonly used by in-school youth in their informal conversations, all participants were interviewed in their local language, Luganda in the Ugandan sample and Dholuo in the Kenyan sample. A face-to-face interview by trained interviewers was chosen to avoid excluding youth not attending school, to ensure both in-depth understanding and consistency within the subsamples. Data were entered directly into handheld computer devices or laptops using dooblo survey software. Interviews took about 50 minutes to an hour on average.

Within the formative research carried out prior to the pilots for the development of the BF intervention, qualitative data were collected through 28 focus group discussions (FGDs) with ALH (aged 10 to 19), health care providers and caregivers, to assess the SRH and broader psychosocial needs of adolescents living with HIV [29]. These data were used in addition to the results of the pre-tests (see above ‘Development of the Eastern African version of the KIDSCREEN and translation into Dholuo and Luganda’) in the current study to validate the results of the confirmatory factor analysis as described below.

### Data Analysis

To explore the data we used SPSS version 16. All items of the KIDSCREEN were re-coded to obtain positive wording so that higher scores indicated higher HRQOL. When confirmatory factor-analytic measurement models (CFA) were estimated with Mplus version 6.1, the MLM estimator was used to correct for the non-normality of our data. The adequacy of the different models is based on Root Mean Square Error of Approximation (RMSEA) and the Comparative Fit Index (CFI). An RMSEA lower than 0.06 (0.08) and a CFI higher than 0.95 (0.90) were considered to indicate an excellent (adequate) fit between the model and the data [30].

The twofold objectives were attained using a two step procedure. To assess the reliability of an Eastern African version of the KIDSCREEN, all three versions (KIDSCREEN-52, -27 and -10) were fitted to the data with CFA. To determine the most suitable measure for resource-constrained settings the three versions of the adapted KIDSCREEN were compared. An adaptation was made based on this result and the qualitative findings in order to develop a final HRQOL measurement instrument.

## Results

### Sample Characteristics

The adapted questionnaire was administered between February and April 2011 to 582 HIV-positive adolescents (aged 13–17) in Kenya (Kisumu, Gem and Asembo: n = 283) and Uganda (Kampala: n = 299). In the Kenyan sample, boys (n = 141) and girls (n = 142) were equally distributed, whereas the Ugandan sample consisted of more female (n = 177) than male (n = 122) adolescents. The mean age of participants in our study was 14.7 years. Results revealed that the average HRQOL of HIV-positive adolescents was above the neutral point of the scale, indicating good HRQOL. Boys scored slightly higher than girls in each domain. However, significant gender differences were only found in relation to *physical well-being*, *peers* and the overall *HRQOL* score.

**Figure 1:**
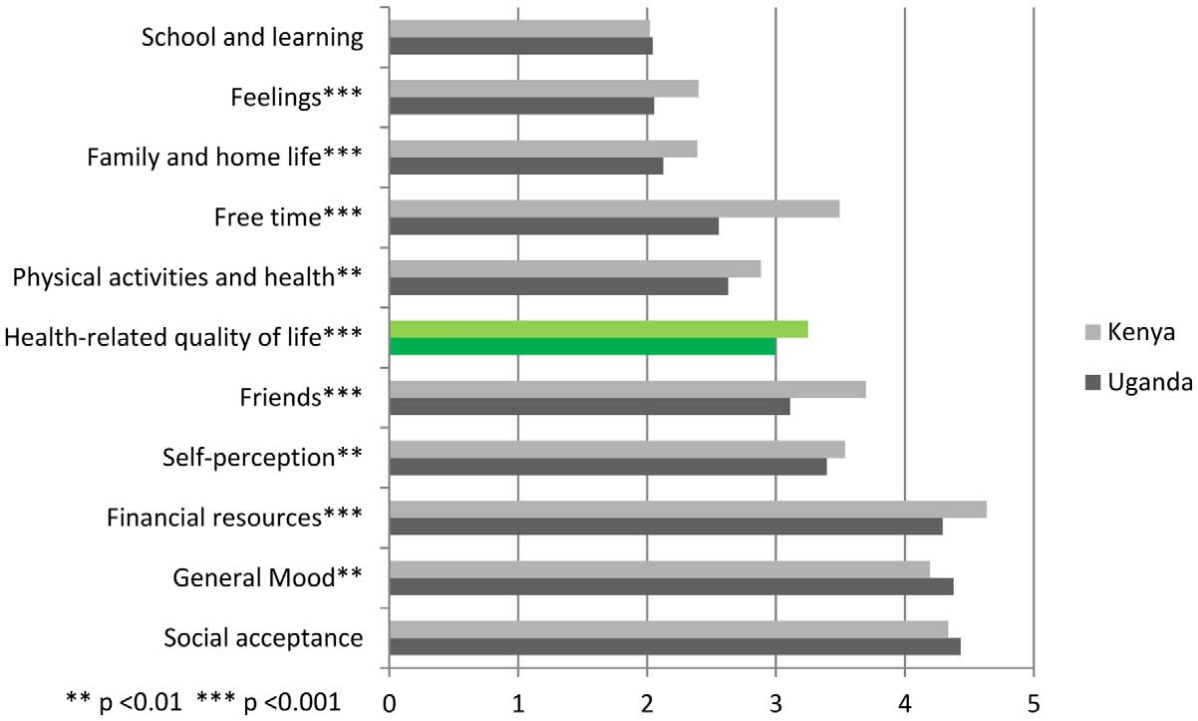
Sum scores of the translated KIDSCREEN-52 in Kenya and Uganda (t-test). Figure 1 compares the average sum scores of Kenyan adolescents on the ten domains of HRQOL, with the scores of their Ugandan counterparts. It can be noted that Kenyan adolescents score slightly higher in every domain, except for *general mood* and s*ocial acceptance*. A comparable score between the two countries can be found for *school and learning*.

Figure 1 offers a graphic presentation of the differences in HRQOL scores of adolescents from Kenya and Uganda. Kenyan HIV-positive adolescents score highest on every domain, except for *general mood* and *social acceptance*. A comparable score between the two countries can be found for *school and learning*.

### Examination of the KIDSCREEN Measurement Models

When assessing the reliability of the HRQOL scale, all three KIDSCREEN versions were fitted to the data with CFA for both countries separately. In accordance with the original factor structure as developed in Europe, a ten, five and single factor structure was fitted to the data for the KIDSCREEN-52, -27 and -10 respectively. In what is to follow, we present the results of the CFA of the three KIDSCREEN models without any adjustments.

#### Original KIDSCREEN-52 questionnaire

CFA on the original KIDSCREEN-52 revealed the fit of the Dholuo version (RMSEA: 0.039; CFI: 0.86) and the Luganda version (RMSEA: 0.048; CFI 0.85) to the data. Four items were below the 0.4 level in the Dhuolo version (questions 19, 20, 29, 50), whereas five items did not reach the recommended 0.4 threshold in the Lugandan model (questions 1, 7, 21, 22, 23). All factor loadings were significant (p<0.001) except for item 20 ‘did you feel happy with your clothes?’ in the Dhuolo model. The cronbach’s alpha of all factors of the latter model was above 0.70, with the exception of the factors *social acceptance* and *self-perception*. The alpha of the latter factor was also in the Lugandan model below 0.70.

#### Original KIDSCREEN-27 questionnaire

A CFA was performed to assess the model fit of the KIDSCREEN-27 (RMSEA Dholuo: 0.056; CFI Dhuolo: 0.83; RMSEA Luganda: 0.071; CFI Luganda: 0.817). All factor loadings were significant with five items below the recommended 0.4 value in both versions (questions Dholuo version: 13, 14, 17, 35, 36; Luganda version: 1, 14, 17, 35, 36). The cronbach’s alpha of *general mood and feelings* and *family and free time* was in the Dhuolo version slightly below 0.70, whereas the cronbach’s alphas of all five Lugandan factors were above 0.75.

#### Original KIDSCREEN-10 questionnaire

Neither translated versions of the KIDSCREEN-10 fitted the data, with CFI (Dholuo: 0.72; Luganda: 0.74) and RMSEA (Dholuo: 0.10; Luganda: 0.11) being outside the range of the recommended values. All factor loadings were significant (p<0.001), with the exception of question 13 ‘did you feel sad?’ and question 17 ‘did you feel lonely?’ in the Dholuo version. Three items of this translation were below the 0.40 value (questions 13, 17, 24), while two items of the Lugandan version did not reach this 0.40 threshold (questions 13, 17). The cronbach’s alpha of the latter version was 0.73, whereas the alpha for the former version was lower than the 0.70 threshold.

### Comparison between the Models

Table 2 shows a comparison between the three versions of the Eastern African KIDSCREEN in its original factor structure. The three versions revealed a model fit below the 0.90 CFI boundary line and/or above the 0.06 RMSEA boundary line. The factor loadings of all models were highly significant, except for two items of the Dholuo-10 and one item in the Dhuolo-52 version. However, in none of the models were all factor loadings above the recommended 0.40 threshold. All the cronbach’s alphas of the Lugandan KIDSCREEN-27 and -10 were above 0.70.

**Table 2:** Comparison of the three translated KIDSCREEN versions in Dhuolo and Luganda.

	Dholuo - Kenya			Luganda - Uganda		
	−52	−27	−10	−52	−27	−10
Model fit: RMSEA ≤0.05; CFI ≥0.90	✗	✗	✗	✗	✗	✗
All Factor loadings >0.40	✗	✗	✗	✗	✗	✗
Significant factor loadings (p<0.001)	✗	✓	✗	✓	✓	✓
Cronbach’s alpha of all factors >0.70	✗	✗	✗	✗	✓	✓

### Final HRQOL Measurement Instrument

Because none of the original KIDSCREEN scales is without any adjustments suitable for the Kenyan and Ugandan setting, we propose to use the translated KIDSCREEN-27 as the best suitable HRQOL measure. This KIDSCREEN version scores relatively better than the other versions as shown in table 2. Furthermore, this version requires less time to administer than the extensive 52 version, but captures nonetheless all the important aspects of HRQOL. However, further adjustments of the KIDSCREEN-27 were required. To interpret the findings of the KIDSCREEN-27 in a culturally appropriate way, we utilized the results of the pilot interviews complemented with the findings of the formative study. We subsequently developed a model based on those results and the modification indices.

**Figure 2:**
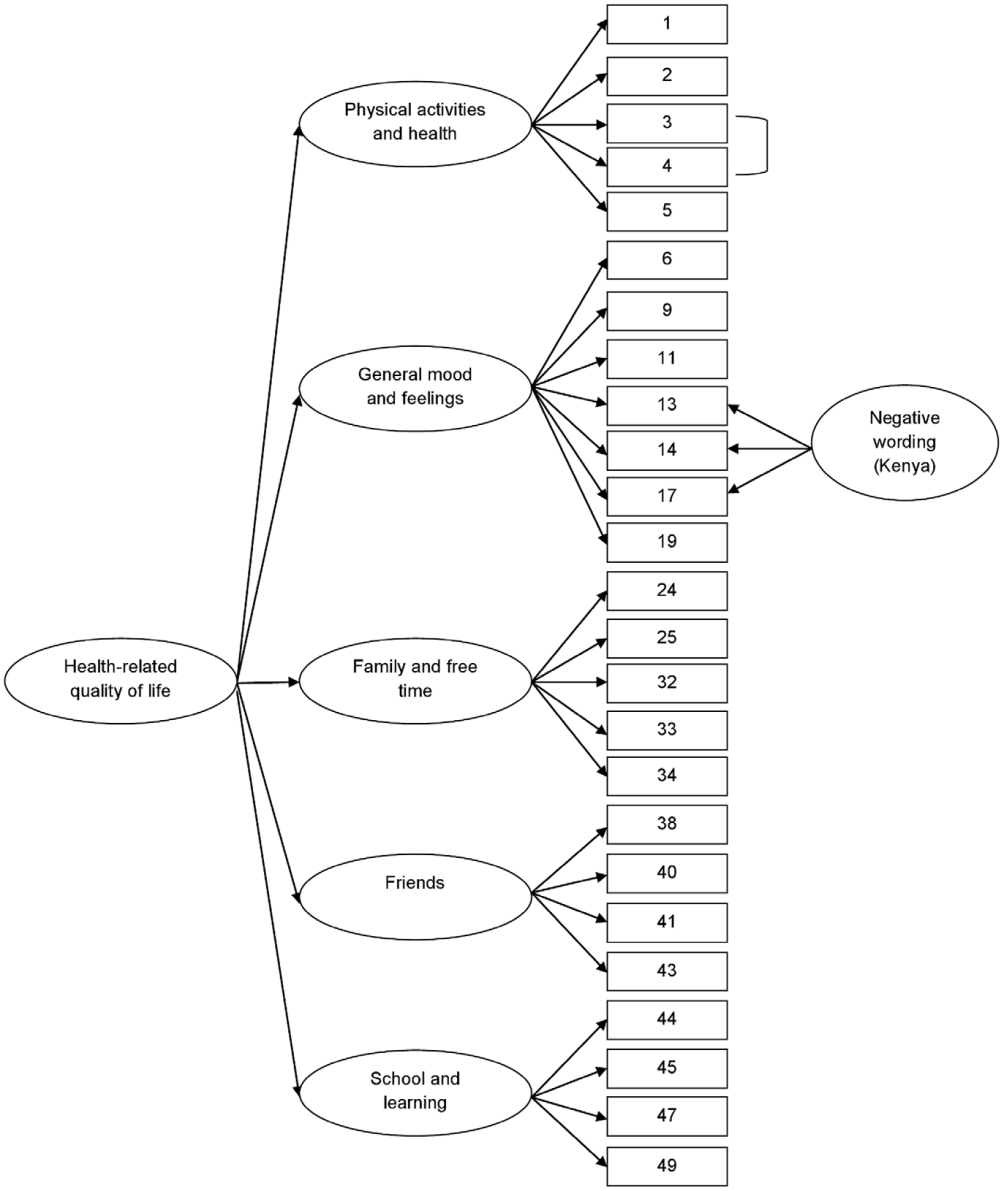
Measurement model of the final HRQOL instrument. Figure 2 offers a graphic representation of the factor structure of our final Dhuolo HRQOL instrument. With the exception of the negative wording factor, this figure also represents the final Lugandan HRQOL measurement instrument. The corresponding question of each number in the rectangle can be found in Table 1. The ellipses represent the method factor (*negative wording*), five first-order factors (*physical activities and health; general mood and feelings; family and free time; friends; school and learning*) and the second-order factor (*health-related quality of life*).

To assess the relation between the first order latent factors obtained from the preliminary CFA, a second-order confirmatory factor-analytic measurement model was fitted to the data, in order to develop a general HRQOL factor. Based on the low factor loadings of the financially related items, which is confirmed by qualitative findings, we omitted both financially related items (items 35 and 36) in our final HRQOL measure. The pilot revealed that adolescents did not usually have their own money at their disposition. Given that adolescents in our study sample were from socioeconomically disadvantaged backgrounds, they did not receive money from their caregivers for any extra expenses apart from what their caregivers provided for them. Furthermore, the error terms of the item 3 (‘were you physically active (e.g. running around, physical exercises, sports))?’ and item 4 (‘were you able to do these physical exercises well?’) of the *physical activities and health* factor were correlated, suggesting that these items are measuring the same underlying construct. In the original KIDSCREEN-27 the factor loadings of the only three negatively worded items were below the 0.40 threshold in the Dhuolo version (i.e. question 13 (0.260), 14 (0.270) and 17 (0.204)). The factor loadings of the two latter Lugandan items were also below the recommended 0.40 value: question 14 (0.302) and 17 (0.396). These findings indicate that the introduction of a negative wording factor in the model might be required.

#### Dholuo version (Kenya)

Adding a negative wording factor to the second-order factor solution improved the loadings of the three Dhuolo negatively worded items (question 13 (0.575), question 14 (0.556), question 17 (0.445)). As indicated by CFI (0.93) and RMSEA (0.039), a good model fit was found. Moreover, all factor loadings were above 0.40 and significant, ranging from 0.421 (question 24) to 0.924 (factor: *physical well-being*). All cronbach’s alphas of the factors were above 0.70, with the exception of the negative factor (α = 0.69). The second-order factor had an alpha value of 0.84. Figure 2 offers a graphic representation of the measurement model of our final Dhuolo HRQOL instrument.

#### Luganda version (Uganda)

Addition of the negative wording factor resulted in worse loadings for the three negative items (question 13 (0.396), question 14 (0.267), question 17 (0.359)). Hence, the final Lugandan model (CFI: 0.90; RMSEA: 0.052) does not contain this method factor. Consequently, two factor loadings remained below the 0.40 boundary line (question 14 (0.302) and 17 (0.396)). All cronbach’s alphas of the factors were above 0.75, with an alpha value of 0.87 for the second-order factor. Figure 2 illustrates as well the Lugandan version of our final HRQOL measure, with the exception of the negative wording factor.

## Discussion

The first objective of this study was to assess the reliability of an Eastern African adaptation of a European HRQL scale for adolescents (KIDSCREEN). Our results indicate that the Dholuo and Lugandan translation of the KIDSCREEN-52, -27 and -10 are without adjustments not suitable for measuring HRQOL of HIV-positive adolescents.

The second objective of this study was to determine which version of the KIDSCREEN is most suitable for measuring HRQOL of adolescents in low-resource settings. Based on the CFA results, the most suitable HRQOL measure is the adaptation of the KIDSCREEN-27. Given that health care personnel work with limited resources, especially in settings with high HIV prevalence, this version is a brief and reliable measurement instrument that is nevertheless based on a comprehensive HRQOL concept. The introduction of a negative wording factor was required for the Dholuo model. Such a method effect may reflect a particular response style, which was found to be more suitable for this specific group of respondents. This result demonstrates the importance of evaluating and correcting for wording effects when examining the factor structure [31]. It is unclear which differences between our two sub-populations may have accounted for the fact that introduction of this factor was required in the Dholuo and not in the Lugandan version. Further research would be required to explore this, but it may point to the high heterogeneity between ethnic groups.

The quantitative CFA results were interpreted in more depth through the qualitative findings (pilots and formative study) aimed at developing a socially and culturally acceptable adaptation of the KIDSCREEN-27. Using qualitative research in instrument adaptation based on a patient-centered approach, results in a tool likely to have more applicability and validity for its users [15]. Furthermore, this multilingual instrument is applicable for cross-cultural research, since it has been translated in a culturally sensitive way. Such an instrument is preferable to a scale which is initially developed in a single culture and then translated without adjustments, as previous research shows that such instruments are unlikely to reflect good semantic and conceptual equivalence in other languages [15]. Nevertheless, not all settings in Sub-Saharan Africa are alike. Additional studies to adapt and test this measurement tool in other low-resource settings with high HIV prevalence are needed in order to yield a cross-cultural instrument.

The limitations of our study need to be acknowledged. The study sample is not fully representative, since respondents were recruited from sites that provide HIV care and support. Consequently, the results cannot be generalized to the whole population of ALH in the study regions, such as for those who are not yet in care. Additional studies need to be conducted to test the validity and reliability of the model on representative data. We could not integrate a medical assessment in the context of this study, and no other HRQOL scales were included in the survey. Further work is needed to validate our final HRQOL instrument for HIV-positive adolescents. Although our final HRQOL measurement instrument is based on a comprehensive definition of quality of life, some HIV-specific challenges potentially impacting on quality of life are missing, such as social isolation or HIV-related stigma. We hypothesize that with increasing access to antiretroviral treatment HRQOL of adolescents may improve, but the social consequences of HIV may continue to impact on overall quality of life of HIV-infected adolescents. It is therefore important to offer an instrument to children and adolescents living with HIV to self-report on their own health status, impact of disease and treatment, social, emotional, cognitive and role functioning.

In conclusion, this instrument can be useful to identify problematic areas and subsequently monitor specific interventions to improve outcomes in these HRQOL domains. Our study shows that the adapted version of the KIDSCREEN can be used as a reliable self-reported instrument for assessing HRQOL in adolescents living with HIV.
